# Screening of lactic acid bacteria strains isolated from Iranian traditional dairy products for GABA production and optimization by response surface methodology

**DOI:** 10.1038/s41598-023-27658-5

**Published:** 2023-01-09

**Authors:** Mohammad Reza Edalatian Dovom, Mohammad Bagher Habibi Najafi, Paria Rahnama Vosough, Neda Norouzi, Seyyed Javad Ebadi Nezhad, Baltasar Mayo

**Affiliations:** 1 Department of Food Science and Technology, Faculty of Agriculture, Ferdowsi University of Mashhad (FUM), Mashhad, Iran; 2 Instituto de Productos Lácteos de Asturias (IPLA-CSIC), 33300-Villaviciosa, Asturias, Spain

**Keywords:** Biotechnology, Molecular biology, Microbiology, Bacteria

## Abstract

A total of 50 lactic acid bacteria (LAB) isolates from Iranian traditional dairy products (Motal and Lighvan cheeses, and artisanal yogurt) were screened for gamma-aminobutyric acid (GABA) production. Firstly, a rapid colorimetric test was performed to evaluate the glutamate decarboxylase (GAD) activity among the LAB isolates examined. Thin layer chromatography (TLC) was then performed on selected strains to identify isolates with high/moderate GABA producing capacity, and a GABase micro-titer plate assay was employed to quantify GABA. Finally, two *Lactococcus* (*Lac.*) *lactis* strains were selected for GABA production optimization via Response Surface Methodology (RSM) following Central Composite Design (CCD). Forty-one out of the 50 isolates showed GAD activity according to the colorimetric assay. Eight isolates displayed strong GAD activity, while nine showed no activity; low to moderate GAD activity was scored for all other isolates. GABA production was confirmed by TLC in all isolates with high GAD activity and in four selected among isoaltes with moderate activity. Among the *Lactococcus* strains tested, *Lac. lactis* 311 and *Lac. lactis* 491 were the strongest GABA producers with amounts of 3.3 and 1.26 mM, respectively. These two strains were subjected to GABA production optimization applying RSM and CCD on three key variables: Monosodium glutamate concentration (MSG) (between 25 and 150 mM), incubation temperature (between 25 and 37 °C), and pH (between 4.0 and 5.0). Optimal conditions for GABA production by *Lac. lactis* 311 and *Lac. lactis* 491 of temperature, pH and MSG concentration were, respectively, 35.4 and 30 °C, pH 4.5 and 4.6, and MSG concentration of 89 and 147.4 mM, respectively. Under the above conditions, the amount of GABA produced by *Lac. lactis* 311 and *Lac. lactis* 491 was 0.395 and 0.179 mg/mL, respectively. These strains and the optimal culture conditions determined in this study could be used for the biotechnological production of GABA or applied in food fermentations for the development of naturally GABA-enriched foods.

## Introduction

Lactic acid bacteria (LAB) are among the most essential and common Gram-positive bacteria which are not only widely distributed in nature and naturally exist in traditional fermented foods, but also extensively involved in industrial food fermentations due to having a reputation of being “Generally Recognized As Safe” (GRAS). The use of pure cultures of lactic acid bacteria for fermentation of cucumbers, cabbage, olives, and other products has been explored for several decades with varying degrees of success. However, at present pure cultures are exploited only on a limited commercial scale for these commodities^1–4^. LAB also offer special functions that are considered beneficial, such as antioxidant and antimicrobial activities, as well as the formation of bioactive compounds such as, among others, peptides, organic acids, and short-chain fatty acids^5–9^. Because of their significant beneficial effects, there are currently noteworthy researches on LAB and attract many industrial interest^10^. Gamma-aminobutyric acid (GABA) is considered as one of the well-known bioactive compounds; it is a four-carbon, non-protein amino acid formed by different organisms including microorganisms, plants, and animals^3,11,12^. This amino acid-derived compound serves as the main inhibitory neurotransmitter in the mammalian central nervous system and, as such, it leds to hypotension, contributes to gut-to-brain signaling and has tranquilizer effects^7,13–16^. Several studies show that GABA contributes to other physiological functions, such as regulating of sleeplessness and depression, enhancing the plasma level of growth hormone, controlling the quorum-sensing systems by acting as a signal molecule between eukaryote cells and pathogens, reducing the inflammation in rheumatoid arthritis^10,17–19^. As a bioactive compound, the application of GABA varies from pharmaceuticals to functional fermented foods^20^. Therefore, it is an ever-growing demand for highly effective GABA biosynthesis, and hence, substantial efforts have been made in this field. Biological methods applying microorganisms are more promising; thus many GABA-enriched products are obtained by fermentation^21–25^. Biosynthesis of GABA from glutamate occurs by the action of the glutamate decarboxylase (GAD), a pyridoxal 5-phosphate-dependant enzyme (EC 4.1.1.15) that is responsible for the conversion of L-glutamate into GABA^3,24^. The wide distribution of GAD among eukaryotes (plants, animals, and fungal strains) and prokaryotes has been reported by several studies^26,27^. However, LAB, besides all beneficial and technological properties, have been introduced as the most promising group of microorganisms in which they are capable of producing a high level of GABA due to high GAD activity^28^. The effort to screen for new GABA-producing LAB still attracts attention, though numerous strains have already been isolated and characterized. Thus, it seems that further research on the isolation and characterization of GABA-producing LAB is demanded to provide novel starters for the nutraceutical and functional fermented foods industry^10^. The diversity of new GABA-enriched foods ranges from cereal-based to dairy-fermented products, including sourdough, bread, cheese, fermented tea and vegetables, traditional Asian fermented foods, and dairy and soy products^1,29^. As mentioned above, by further screening, the isolation sources should be diversified as much as possible, including traditional fermented foods to achieve new GABA-producing LAB strains. This leads to a more comprehensive application area and higher flexibility of starter cultures^11^. According to the literature, raw milk cheeses have been identified as a valuable source of microbial biodiversity and new LAB strains with health-promoting properties^14^. Though currently, there are several different types of researches and industrial interest in the biological examination of the potential of traditional dairy LAB^30–32^. So far, no GABA-producing LAB have been reported from Iranian traditional fermented foods. Recently Azizi et al. (2017) have claimed that most of the isolates from Motal cheese could be used as starters or adjunct-starters in novel fermented functional foods. Besides having antimicrobial properties^33^, the production of bioactive compounds, such as GABA, could potentially substantiate the functionality claimed for such cultures.

Hence, the present study aims to screen for GABA-producing LAB from various Motal and Lighvan cheeses and yogurt samples which are among the preeminent and most appreciated traditional Iranian dairy products.

## Materials and Methods

### Chemical and Media

The commercial GABase kit and all chemicals such as NADP + (Nicotinamide adenine dinucleotide phosphate), α-ketoglutarate, monosodium glutamate (MSG), Triton X-100®, and GABA were purchased from Sigma-Aldrich (St. Louis, MO, USA). M17 and de Man, Rogosa, and Sharpe (MRS) broth media were obtained from Quelab (Quelab Laboratories Inc., Canada).

### LAB strains and growth conditions

A total of 50 LAB isolates previously isolated from Iranian traditional dairy products (Motal cheese^33^, Lighvan cheeses^31,34^, and Iranian yogurt^35^), identified using molecular techniques and 16S rRNA gene sequencing, were screened for GABA production (Table 1). All examined isolates, including lactobacilli (*Lactobacillus, Lactiplantibacillus*, and *Levilactobacillus*) (27 isolates), *Lactococcus* (20 isolates), and *Streptococcus* (3 isolates), were kept frozen in MRS (lactobacilli) or M17 (lactococci and streptococci) broth with 15% glycerol at -80 °C. Pre-cultures were made by adding 20 µL of stock culture to 10 mL fresh medium. Lactobacilli strains were grown in MRS medium, while M17 medium was used for lactococci and *Streptococcus* (*St.*) *thermophilus* strains. Based on the types, incubations were done at 30 or 37ºC for 24–48 h under aerobic or anaerobic conditions.

**Table 1:** GAD activity, Colorimetric results and GABA contents (mg/ml) of lactic acid bacteria isolated from Iranian traditional dairy products.

No	Strain Code	Species	Source	GAD activity	Colorimetric results	GABA content (mg/ml)
1	43	*St.thermophilus*	Iranian yoghurt	+ + + +	Blue	0.02 ± 0.003
2	74	*St.thermophilus*	Iranian yoghurt	+ + +	Greenish blue	0.01 ± 0.001
3	78	*St.thermophilus*	Iranian yoghurt	+ + +	Greenish blue	0.01 ± 0.001
4	4	*Lb. delbrueckii* ssp*. bulgaricus*	Iranian yoghurt	+ + +	Greenish blue	0.02 ± 0.001
5	51	*Lb. delbrueckii* ssp*. bulgaricus*	Iranian yoghurt	+ +	Green	ND
6	62	*Lb. delbrueckii* ssp*. lactis*	Iranian yoghurt	+ +	Green	ND
7	64	*Lb. delbrueckii* ssp*. lactis*	Iranian yoghurt	+ +	Green	ND
8	65	*Lb. delbrueckii* ssp*. lactis*	Iranian yoghurt	+ +	Green	ND
9	67	*Lb. delbrueckii* ssp*. lactis*	Iranian yoghurt	+ + +	Greenish blue	0.04 ± 0.054
10	69	*Lb. delbrueckii* ssp*. lactis*	Iranian yoghurt	+ + +	Greenish blue	0.07 ± 0.003
11	73–1	*Lb. delbrueckii* ssp*. lactis*	Iranian yoghurt	+	Greenish yellow	ND
12	73–2	*Lb. delbrueckii* ssp*. lactis*	Iranian yoghurt	+	Greenish yellow	ND
13	76	*Lb. delbrueckii* ssp*. lactis*	Iranian yoghurt	+ + +	Greenish blue	0.06 ± 0.004
14	86	*Lb. delbrueckii* ssp*. lactis*	Iranian yoghurt	+	Greenish yellow	ND
15	97	*Lac. lactis* ssp. *lactis*	Fresh Lighvan milk	+	Greenish yellow	ND
16	206	*Lac. lactis* ssp. *lactis*	Fresh Lighvan milk	+ +	Green	ND
17	208	*Lac. lactis* ssp. *lactis*	Fresh Lighvan cheese	+	Greenish yellow	ND
18	219	*Lac. lactis* ssp. *lactis*	Fresh Lighvan cheese	+	Greenish yellow	ND
19	220	*Lac. lactis* ssp. *lactis*	Fresh Lighvan milk	+ +	Green	ND
20	261	*Lac. lactis* ssp. *lactis*	Lighvan Curd	+ + + +	Blue	0.04 ± 0.002
21	290	*Lac. lactis* ssp. *lactis*	Lighvan Curd	+ +	Green	ND
22	311	*Lac. lactis* ssp. *lactis*	Lighvan Curd	+ + + +	Blue	0.34 ± 0.006
23	412	*Lac. lactis* ssp. *lactis*	Fresh Lighvan milk	+ +	Green	ND
24	433	*Lac. lactis* ssp. *lactis*	Lighvan Curd	+ +	Green	ND
25	447	*Lac. lactis* ssp. *lactis*	Lighvan Curd	+ +	Green	ND
26	449	*Lac. lactis* ssp. *lactis*	Lighvan Curd	+ +	Green	ND
27	451	*Lac. lactis* ssp. *lactis*	Fresh Lighvan milk	+ +	Green	ND
28	454	*Lac. lactis* ssp. *lactis*	Fresh Lighvan milk	+ + + +	Blue	0.08 ± 0.001
29	466	*Lac. lactis* ssp. *lactis*	Fresh Lighvan milk	+ +	Green	ND
30	473	*Lac. lactis* ssp. *lactis*	Lighvan Curd	+ +	Green	ND
31	485	*Lac. lactis* ssp. *lactis*	Lighvan Curd	+	Greenish yellow	ND
32	487	*Lac. lactis* ssp. *lactis*	Fresh Lighvan milk	+ +	Green	ND
33	491	*Lac. lactis* ssp. *lactis*	Fresh Lighvan milk	+ + + +	Blue	0.13 ± 0.017
34	506	*Lac. lactis* ssp. *lactis*	Lighvan Curd	+ +	Green	ND
35	M2	*Lb. brevis*	Motal cheese	+ + + +	Blue	0.12 ± 0.004
36	M3	*Lb. brevis*	Motal cheese	–	Yellow	ND
37	M4	*Lb. brevis*	Motal cheese	+ + + +	Blue	0.36 ± 0.015
38	M5	*Lb. brevis*	Motal cheese	+ +	Green	ND
39	M6	*Lb. brevis*	Motal cheese	–	Yellow	ND
40	M7	*Lb. brevis*	Motal cheese	–	Yellow	ND
41	M8	*Lb. brevis*	Motal cheese	–	Yellow	ND
42	M9	*Lb. brevis*	Motal cheese	–	Yellow	ND
43	M11	*Lb. brevis*	Motal cheese	+ +	Green	ND
44	M12	*Lb. brevis*	Motal cheese	+ + + +	Blue	0.18 ± 0.006
45	M13	*Lb. brevis*	Motal cheese	–	Yellow	ND
46	M15	*Lb. casei*	Motal cheese	–	Yellow	ND
47	M16	*Lb. plantarum*	Motal cheese	+ +	Green	ND
48	M17	*Lb. plantarum*	Motal cheese	–	Yellow	ND
49	M18	*Lb. plantarum*	Motal cheese	+ +	Green	ND
50	M19	*Lb. plantarum*	Motal cheese	–	Yellow	ND

(+ +  + +): High,(+ + +): Medium, (+ +): Low, ( +) : very Low, (–):No GAD.

ND: not determine.

### Qualification screening of LAB isolates

#### Determination of GAD activity

Glutamic acid decarboxylase (GAD) assay is a rapid colorimetric test used to assess the GABA-producing potential among LAB isolates. Sample preparation for GAD activity determination was done as described by Lacroix et al.^36^. An overnight grown culture of each isolate (5 mL) was washed with 0.9% (w/v) NaCl solution and centrifuged at 5000 × g (20 min, 25 ºC). Cells were suspended in 500 µL of GAD reagent solution (pH = 4), which consists of L-glutamic acid (1 g), Triton X-100 (300 µL), NaCl (90 g), and bromocresol green (0.05 g) in 1 L deionized water. After anaerobic incubation for 4 h at 37ºC, GAD activity was visually examined by color change. The development of a greenish or blueish color was considered as low or high GAD activity, respectively. However, no color change (yellow) was considered with no activity^36^.

#### Thin layer chromatography (TLC)

To identify isolates with high and moderate GABA producing ability, a TLC assay was performed as described by Lee et al.^37^. Briefly, strains were inoculated in their respective broth medium supplemented with 0.5% monosodium glutamate (MSG) and incubated at their optimal temperature for 24 h. To test the presence of GABA, supernatants were collected by centrifugation (8000 × g, 4ºC, 5 min) and then 2 µL of each filtered supernatant was spotted on a TLC plate (Silica gel 60 F254). TLC was conducted using n-butanol:acetic acid:water ratio of 5:2:2 (v/v/v). The silica gel plate was subsequently developed by spraying with a 2% ninhydrin solution and heating at 105ºC for 5 min.

### DNA extraction and strain typing by rep-PCR

Total genomic DNA was extracted from GABA-producing isolates using a commercial kit (High Pure PCR Template Preparation Kit; Roche, Basel, Switzerland), following the manufacturer’s instructions. To assess the interspecies diversity between isolates, repetitive extragenic profiling PCR (rep-PCR) was done using BoxA2R (5´-ACGTGGTTTGAAGAGATTTTCG-3´) as reported by Koeuth et al.^38^. In brief, reactions were performed in 25 µL volume containing 2 µL purified DNA, 12.5 µL Red master mix (Ampliqon, Odense, Denmark), 9.3 µL Nuclease-free water, and 1.2 µL of the primer. Thermal conditions were as follows: an initial step of denaturation at 95 °C for 5 min, followed by 35 cycles of denaturation and annealing at 94 °C for 29 s, 40 °C for 1 min, and at 68 °C for 8 min; with a final extension at 68 °C for 10 min. PCR products were then electrophoresed using 1.5% agarose (Merck, Darmstadt, Germany) gel at 75 V for 90 min. DNA safe stain (0.75 µL/100 mL) (Sina Clon Bio Science, Tehran, Iran) was used for visualization of the gel and the bands photographed under UV light by Gel Doc System. The similarity of the patterns was expressed by the Simple Matching (SM) coefficient, and the clustering was performed using the Unweighted Pair Group Method with Arithmetic Mean (UPGMA) method, with the Multivariate Statistical Package (MVSP) software version 3.13d^31^.

### GABA determination

Quantitative measurment of GABA in broth medium was done by a GABase microtiter plate assay as described by Tsukatani et al.^39^. Bacterial isolates were grown in MRS broth medium containing 0.5% MSG for 2 days at their optimal growth temperature. The reactive mixture contained 750 mM sodium sulfate, 10 mM dithiothreitol, 1.4 mM NADP + , 2.0 mM α-ketoglutarate in Tris–HCl buffer (80 mM, pH 9.0) and GABA-T (Gamma-aminobutyrate glutamate aminotransferase) (Sigma-Aldrich). The mixture was added to each well (in a 96-well microtiter plate) in a final volume of 90 µL. The initial absorbance was read at 340 nm with a microplate reader (BioTek, ELX 808) before adding a 10 µL sample of a standard GABA solution. The substrate of NADP + turns to NADPH in the presence of GABA and α-ketoglutarate after incubation at 30-37ºC for 1 h. To calculate the GABA content formed by each isolate, the difference between initial and final absorbances at 340 nm was compared to standard curves of calibrated GABA solutions.

### Optimization of GABA production using response surface methodology (RSM)

Optimization of GABA production in MRS medium and studying the interaction of key parameters were done using response surface methodology (RSM). A Central Composite Design (CCD) of RSM was applied to optimize the 3 effective variables: MSG concentration (between 25 and 150 mM), incubation temperature (from 25 to 37 °C), and pH (4.0–5.0). Varying these three independent parameters, a total of 20 experiments for each isolate were designed and performed. The statistical software Design Expert 10.0.7.0 was used for data analysis^12,40^. Laboratory-scale fermentations were set up to investigate optimal parameters for GABA production by selected isolates. Bacterial isolates were inoculated into falcon tubes containing 50 mL MRS broth medium with different concentrations of MSG with initial pH adjustment and incubated at different temperatures for 48 h to reach optimum GABA production. After 48 h of incubation, samples were withdrawn from the each falcon tube to determine GABA content.

### Statistical analysis

To analyze the results related to the potential of GABA production by examined bacteria, Duncan’s one-way analysis of variance (ANOVA) was applied using the SPSS software (ver. 16), while to optimize the conditions of GABA production, RSM with Design Expert 10.0.7.0 software was used.

## Results

### Screening of GAD activity

Total number of LAB isolates and the results of their previous identification^31,33–35^ are presented in Table 1. These isolates include members of lactobacilli (of the genera *Lactobacillus, Lactiplantibacillus*, and *Levilactobacillus*) (27 isolates), *Lactococcus* (20 isolates), and *Streptococcus* (3 isolates).

The GAD activity of the 50 isolates from Iranian traditional dairy products are also summarized in Table 1. The cultures of the isolates *Levilactobacillus* (*Lb.*) *brevis* M2, M4 and M12; *Streptococcus* (*St.*) *thermophilus* 43, and *Lac. lactis* 261, 311, 454, and 491 developed a strong blue color, consistent with a high GAD activity. No color change was observed for the isolates M3, M6, M7, M8, M9, M13, M15, M17, and M19; thus these are considered not to have GAD activity. Other isolates with greenish-blue, green and greenish-yellow color were considered as having medium, low and very low GAD activity, respectively. Fourteen isolates, including all high and some with moderate GAD activity were selected for subsequent experiments. GAD assay results revealed that most of the GAD-positive isolates were isolated from cheese (Lighvan and Motal); whereas, only one isolate from a traditional yogurt exhibited high GAD activity.

### Genetic analysis of isolates

**Figure 1:**
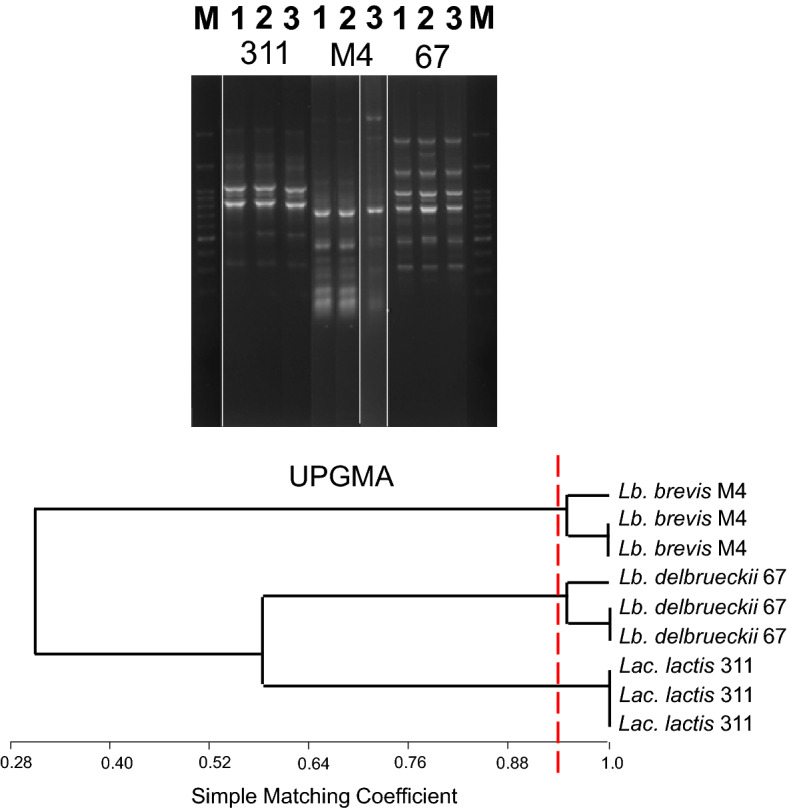
Repeatability of the rep-PCR typing assay with primer BoxA2R after analysing three randomly-selected isolates (311, M4, and 67) in three independent experiments (1, 2, and 3). Below, dendogram of similarity of the patterns obtained clustered by the UPGMA method using the Simple Matching coefficient. The dotted red line indicates the repeatibility considered in this work (94%) to separate isolates from strains. M, molecular weight marker.

**Figure 2:**
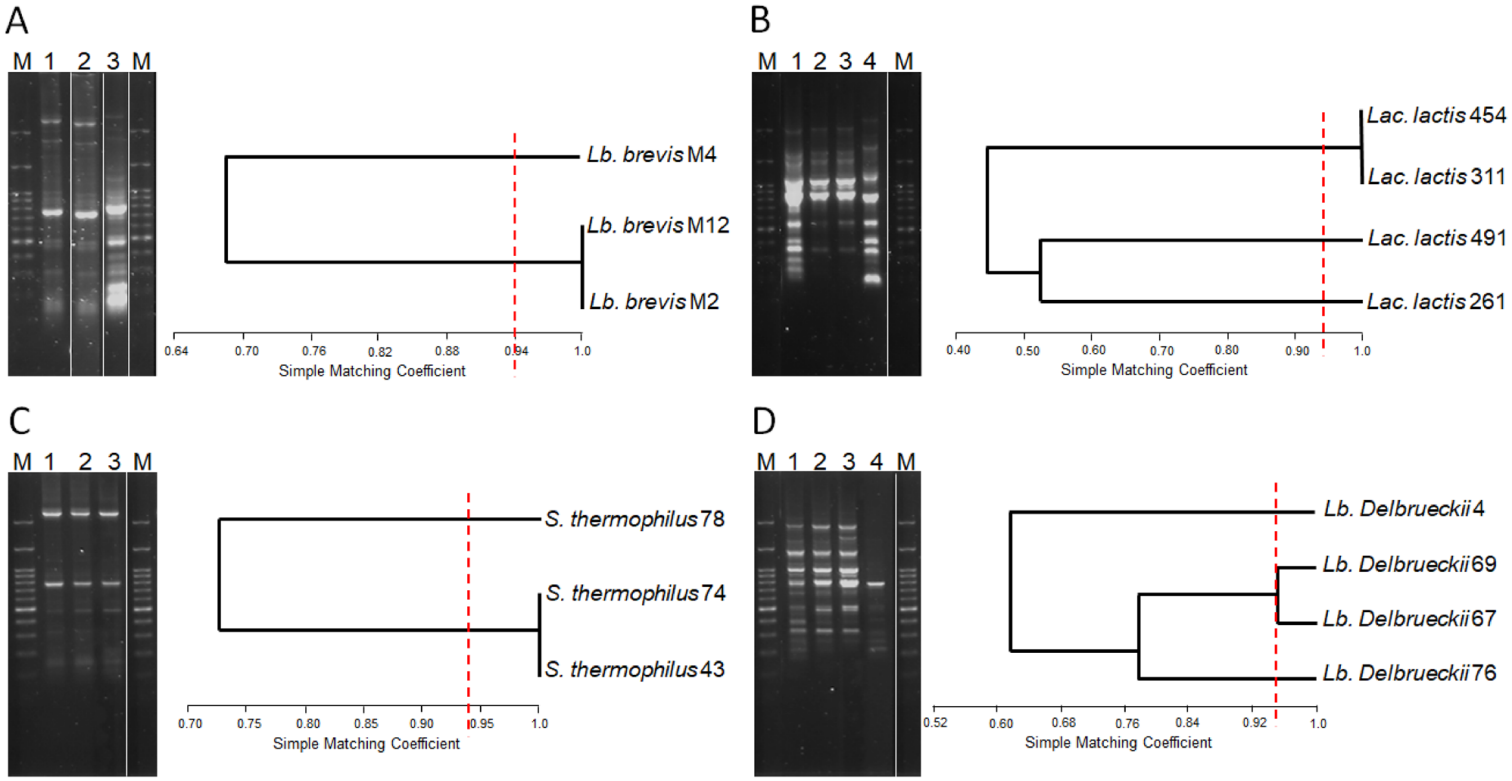
rep-PCR typing profiles obtained with primer BoxA2R for the 14 lactic acid bacteria isolates producing GABA and belonging to the species *Levilactobacillus brevis* (**A**), *Lactococcus lactis* (**B**), *Streptococcus.thermophilus* (**C**), and *Lactobacillus delbrueckii* (**D**). Besides each of the gels, a dendogram of similarity of the different patterns clustered by the UPGMA method using the Simple Matching Coefficient is shown. M, molecular weight marker. Red lines show the repeatibility of the typing method (94%).

Lactobacilli, *Streptococcus* and *Lactococcus* species were targeted by rep-PCR to evaluate the intramolecular diversity among the isolates, as well as to assess whether isolates with GAD activity belonging to the same species were replicates or independent strains. Further, typing of the strains could serve for selecting different strains for GABA production optimization. The different patterns of similarity were clustered using the SM coefficient by the UPGMA method. Given the reproducibility of the assay around 94 (Fig. 1), isolates sharing a percentage of similarity > 94% were considered to belong to the same strain. Figure 2 a, b, c and d are the profiles obtained from 3 isolates of *Lb. brevis*, 4 isolates of *Lac. lactis*, 3 isolates of *St. thermophilus* and 4 isolates of *Lb. delbrueckii. Lb. brevis* M2 and *Lb. brevis* M12 strains showed 100% similarity. *Lac. lactis* 311 and *Lac. lactis* 454 strains had 100% similarity. *St. thermophilus* 74 and *St. thermophilus* 43 also had 100% similarity. These strains can be multiple isolates. *Lactobacillus* (*Lb.*) *delbrueckii* 69 and *Lb. delbrueckii* 67 also showed very high homology. The relation between the intra-species diversity with GABA production in these species showed that GABA production was significantly different between isolates with high similarity and comparable between isolates with low similarity. Unlike other examined strains, there was no significant difference in GABA production in *St. thermophilus* isolates with high and low similarity.

### Confirmation of GABA by thin layer chromatography

**Figure 3:**
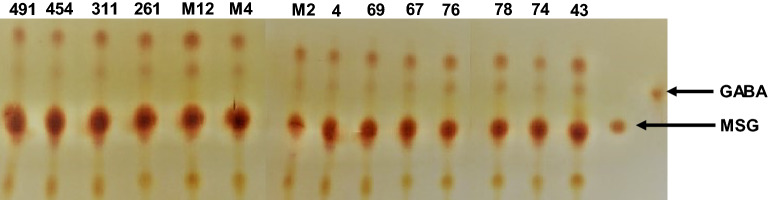
TLC analysis of GABA produced the 14 lactic acid bacteria isolates, as compared to the retention factor (Rf) of a commercial GABA standard (= 0.77 cm).

Isolates that showed no GAD activity by the colorimetric method were excluded from further analyses. Based on the results of GAD activity, we selected 14 isolates corresponding to the species *St. thermophilus* (three), *Lb. delbrueckii* (four), *Lac. lactis* (four), *Lb. brevis* (three) for further studies. The GABA production was confirmed by observing red spots on the TLC plates (Fig. 3). The spot mobility from the culture supernatant of the 14 isolates was consistent with GABA, as a retention factor (Rf) value (0.77 cm) of the sample matched with the GABA standard. Thus, all 14 selected isolates were considered as GABA producers.

GABA’s of strains show the same retention factor (Rf) value as the GABA standard (= 0.77 cm). The retention factor was defined as the ratio of the distance traveled by the center of a spot to the distance traveled by the solvent front.

### Quantitative GABA production measurement

GABA production of selected strains was quantified using a GABase microtiter plate enzymatic determination. As shown in Table 1, GABA production varied widely ranging from 0.01 to 0.36 mg/L (equivalent to 0.12 and 3.52 mM). All *Lb. brevis* isolates from Motal cheese exhibited a high ability to produce GABA (> 1 mM), while the highest GABA concentration was produced by *Lb. brevis* M4. Our findings further revealed that all isolates belonging to *Lac. lactis* have a medium to high GABA producing ability, while all *Lb. delbrueckii* cultures presented a low level of this ability.

### RSM design

Although *Lac. lactis* isolates produced less GABA than the *Lb. brevis* isolates, the former species is the most economically-important starter component in cheese^43^, which prompted us to select two different strain of this species, *Lac. lactis* 311 and *Lac. lactis* 491, for GABA production optimization by RSM methodology. The selected two *Lac. lactis* strains are different strains according to the rep-typing results.As stated in the corresponding section, a CCD (central composite design) was performed based on a single variable factor test at a time and considering three independent variables: MSG concentration (mM), Temperature (°C), and pH. The 20 different combination set ups based on CCD and the corresponding GABA concentration for both predicted and experimental values are listed in Tables 2 and 3, respectively.

**Table 2:** Three independent variables set of 20 experiments were designed and performed at five levels and one repetition for GABA concentration by *Lac. lactis* 311.

Run	Variables			GABA (mg/ml)	
	MSG (X1)	T (X2)	pH (X3)	Experimental value	Predicted value
1	87.5	41	4.5	0.345	0.351
2	25	37	5	0.310	0.308
3	25	37	4	0.340	0.289
4	150	25	5	0.301	0.295
5	87.5	31	4.5	0.389	0.381
6	150	37	5	0.350	0.385
7	150	25	4	0.280	0.260
8	87.5	31	4.5	0.370	0.381
9	150	37	4	0.370	0.358
10	25	25	4	0.193	0.194
11	87.5	31	4.5	0.390	0.381
12	87.5	31	4.5	0.388	0.381
13	192.6	31	4.5	0.390	0.365
14	87.5	20.9	4.5	0.175	0.196
15	5	31	4.5	0.285	0.279
16	87.5	31	4.5	0.380	0.381
17	25	25	5	0.231	0.222
18	87.5	31	4.5	0.400	0.381
19	87.5	31	3.6	0.278	0.281
20	87.5	31	5.3	0.324	0.327

**Table 3:** Three independent variables set of 20 experiments were designed and performed at three levels and one repetition for GABA concentration by *Lac. lactis* 491.

Run	Variables			GABA (mg/ml)	
	MSG (X1)	T (X2)	pH (X3)	Experimental value	Predicted value
1	87.5	31	4.5	0.174	0.172
2	150	37	4	0.163	0.164
3	25	25	5	0.089	0.089
4	150	37	5	0.176	0.176
5	192.6	31	4.5	0.169	0.169
6	87.5	31	5.3	0.144	0.145
7	87.5	41	4.5	0.147	0.147
8	5	31	4.5	0.108	0.108
9	87.5	31	3.6	0.128	0.128
10	87.5	31	4.5	0.172	0.172
11	150	25	5	0.125	0.126
12	87.5	31	4.5	0.173	0.172
13	87.5	31	4.5	0.177	0.172
14	25	37	5	0.133	0.122
15	87.5	31	4.5	0.179	0.172
16	150	25	5	0.127	0.130
17	87.5	31	4.5	0.175	0.172
18	25	25	4	0.083	0.083
19	25	37	4	0.107	0.107
20	87.5	20.9	4.5	0.088	0.088

The interaction effect of these factors on GABA production concentration by *Lac. lactis* 311 is described by the following equation:

1 $$\begin{aligned} {\text{R}} = & 0.{18} + 0.0{23} \times {\text{A}} + 0.0{19} \times {\text{B}} + {5}.{\text{429E}} - 00{3} \times {\text{C}} + {2}.{\text{363E}} - 00{3} \times {\text{AB}} - {1}.{\text{937E}} \\ - 00{3} \times {\text{AC}} + {3}.{\text{863E}} - 00{3} \times {\text{BC}} - 0.0{16} \times {\text{A}}^{{2}} - 0.0{2}0 \times {\text{B}}^{{2}} - 0.0{13} \times {\text{C}}^{{2}} \\ \end{aligned}$$

where R is the concentration of GABA (mg/mL) and A, B and C are coded values of the independent variables, viz., MSG, temperature and pH, respectively.

**Figure 4:**
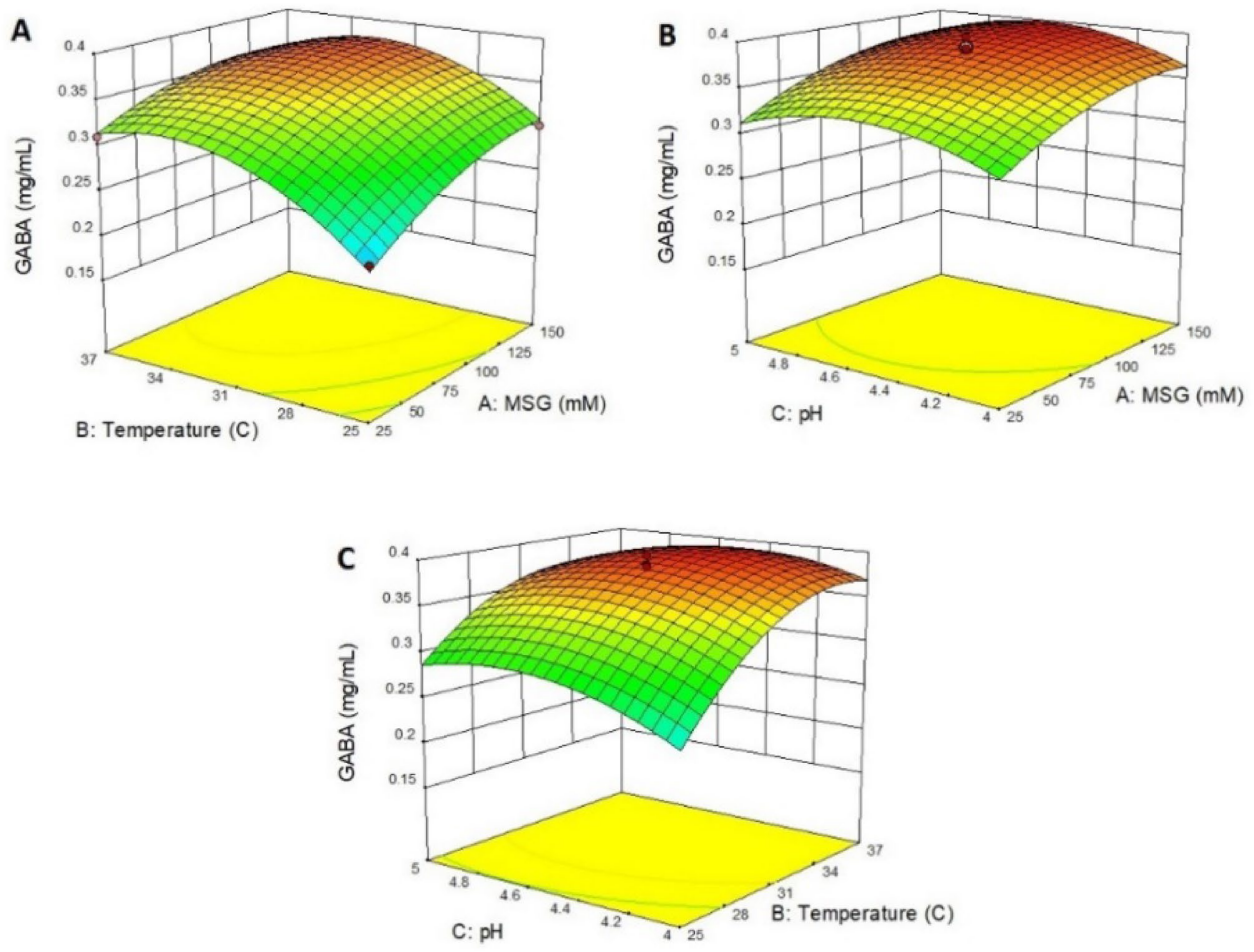
3D plots showing the interaction effect of three variables for GABA production by *Lac. lactis* 311. (**A**) surface-response curve showing the interaction effect of MSG and temperature, keeping pH at its central value; (**B**) 3D curve for the interaction of MSG and pH, keeping temperature T at its central value; (**C**) 3D curve for the interaction of temperature and pH, keeping MSG concentration at its central value.

Three-dimensional surface plots were generated to estimate the effect of the combinations of the independent variables on the GABA production using Design Expert software. The 3D plot in Fig. 4A shows the interaction effect of MSG and temperature, keeping pH at its central level 4.5. According to the 3D plot, predictably, the GABA concentration increased with increasing MSG concentration and the increase of temperature. Nevertheless, in the low MSG concentration, increasing GABA concentration was more sharply compared to a higher concentration. The maximum concentration (3.83 mM) was observed with MSG in concentration of 89 mg/mL and temperature of 35.4 °C.

Figure 4B presents the 3D plot showing the dependency of GABA concentration on MSG concentration and pH, keeping the third factor (temperature) at its central level of 31 °C. The graph predicted that the highest GABA concentration by relevant strain at 35.4 °C was observed with the mid pH (4.5) and MSG concentration (89 mg/ml). However, GABA concentration at lower pH remained approximately constant by increasing MSG concentration. The 3D curve showing the interaction effect of temperature and pH has shown in Fig. 4C. The low GABA concentration was observed with the lowest temperature and lowest pH. The maximum GABA concentration (3.83 mM) was observed with pH (4.5) and temperature (35.4 °C).

Equation 2 explains the interaction effect of the independent variables on GABA concentration by *Lac. lactis* with strain code 491:

2 $$\begin{aligned} {\text{GABA}} = & 0.{39} + 0.0{32} \times {\text{A}} + 0.0{48} \times {\text{B}} + {6}.{\text{314E}} - 00{3} \times {\text{C}} - 0.0{11} \times {\text{AB}} - {7}.{\text{459E}} \\ - 00{4} \times {\text{AC}} - 0.0{14} \times {\text{BC}} - 0.0{19} \times {\text{A}}^{{2}} - 0.0{43} \times {\text{B}}^{{2}} - 0.0{28} \times {\text{C}}^{{2}} \\ \end{aligned}$$

**Figure 5:**
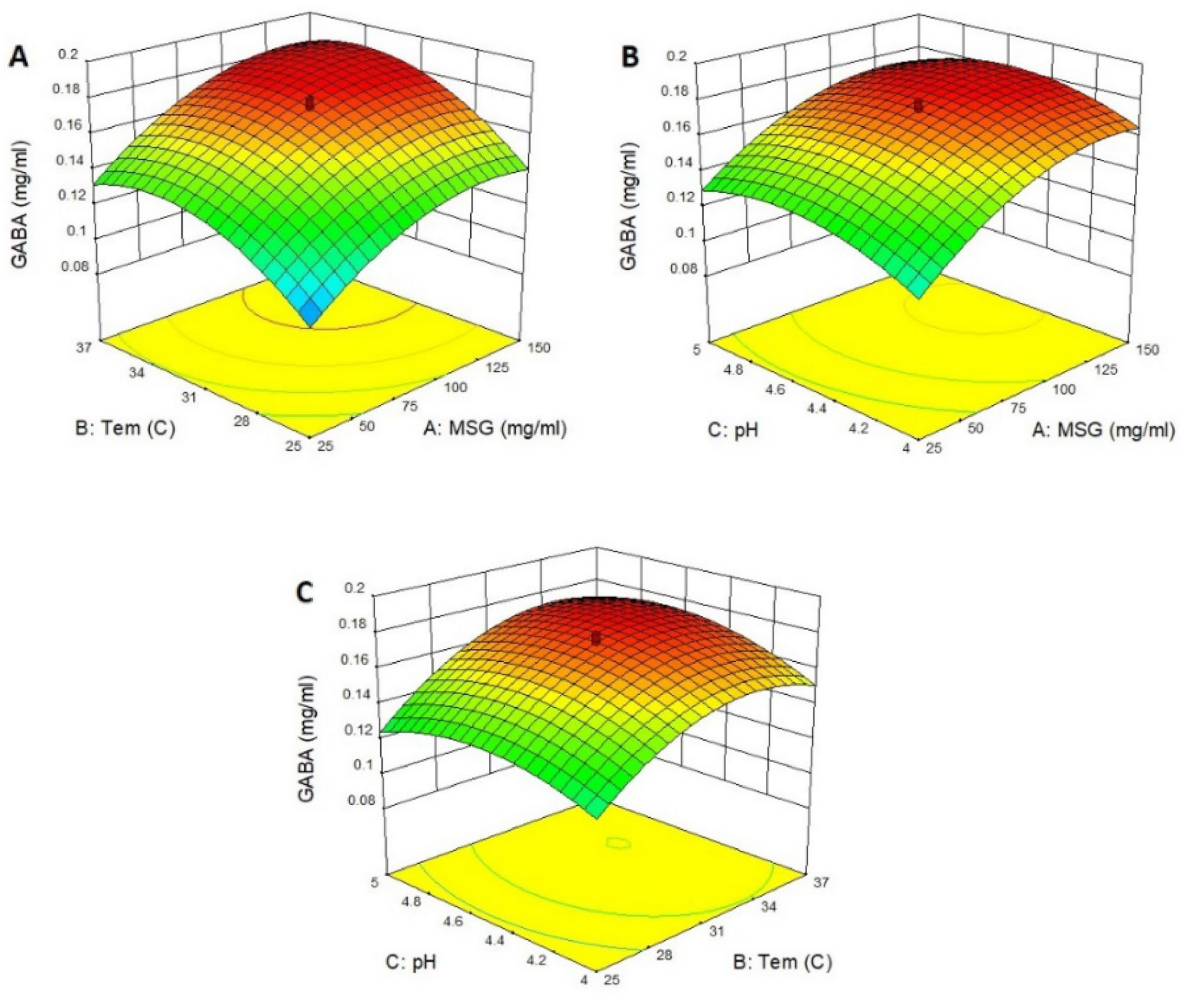
3D plots showing the interaction effect of three variables for GABA production by *Lac. lactis* 491. A, surface-response curve showing the interaction effect of MSG and temperature, keeping pH at its central value; B, 3D curve for the interaction of MSG and pH, keeping temperature T at its central value; C: 3D curve for the interaction of temperature and pH, keeping MSG concentration at its central value.

Figure 5A showed that with increasing the concentration of monosodium glutamate and increasing the temperature, the amount of GABA formed increased. This trend is in agreement with the results reported elsewhere by other authors^41,42^.

An increase in the amount of GABA was observed with increasing MSG and pH to 89 mg/ml and 4.59, respectively, at a constant temperature (Fig. 5B). When the concentration of monosodium glutamate was kept constant, the GABA level increased sharply with increasing temperature, but increased pH led to the production of low amounts of GABA (Fig. 5C). The incubation temperature is another important factor that may affect GABA production. In addition to the stability and biological activity of the enzyme, temperature also affects the thermodynamic equilibrium of the reaction.

### Selection of optimal treatment and validation of model

**Figure 6:**
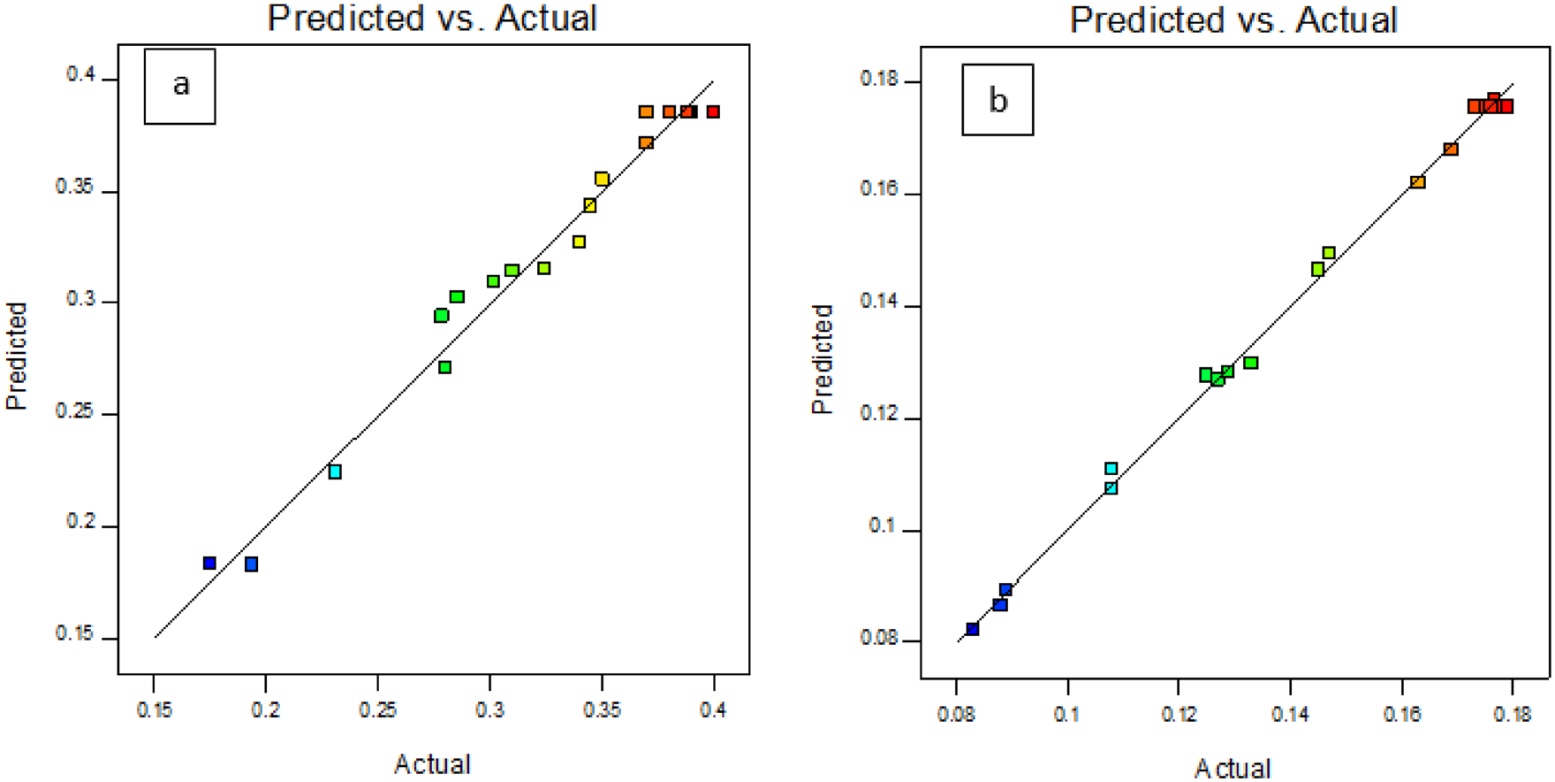
Model validation for *Lac. lactis* 311 (**a**) and *Lac. lactis* 491.

The results of GABA production by two examined strains showed the optimal conditions for GABA production by *Lac. lactis* 311 and *Lac. lactis* 491 had a temperature of 35.4 and 30 °C, a pH of 4.5, 4.6 and a concentration of 89 and 147.4 mM of MSG, respectively. In the above conditions, the amount of GABA production by these two strains was 3.83 and 1.73 mM, respectively. Examination of the results in Fig. 6 shows that the points with a good approximation are on the straight line and the values obtained experimentally are in a good agreement with the values predicted by the model and showed the accuracy of the equation model.

## Discussion

Regarding the Motal cheese isolates in this study, the results are consistent with previously reported levels by Ebadi Nezhad et al. (2020)^44^. GABA production begins in the exponential growth phase and increases near to the stationary phase due to an increased in GAD activity, which agrees with GAD being an intracellular enzyme produced in response to acidic conditions^45^. The nutrient sources required for the GABA production by bacteria include the primary sources of carbon, nitrogen (including GABA substrate, glutamic acid) and minerals^46^.

To avoid the use of replicates from the optimization studies, a strain-level typing scheme by rep-PCR was employed. Knowing intra-species diversity also helps to know how diverse the extend of GABA production is in closely-related and unrelated strains of the same species^10^. As for many other phenotypic traits, large phenotypic variations have occasionally been reported for genetically closely-related LAB strains^47^. Typing of LAB isolates from Koozeh and Lighvan cheese by rep-PCR with BOXA2R primer and typing of GABA-producing LAB isolates with rep-PCR by (GTG)_5_ primer has been previously reported by others^10,31^. As in this work, high intraspecies diversity was reported in the referred studies.

Presumptive detection of GABA production by LAB species by TLC, followed by its confirmative identification and quantification by High-performance liquid chromatography (HPLC) has been previously reported to be a successful strategy by Zhang et al.^48^. These authors reported that the mobility spot of the culture supernatant of BC114 strain was basically consistent GABA standard and could be preliminarily identified as GABA within a negligible margin of error. The measured sample from the fermentation broth (a complex matrix), may also result in incomplete agreement with pure GABA standards^48^. The strain under investigation, *Lb. plantarum* BC114, showed the highest GABA production as measured by HPLC, which reached a value of 1.52 ± 0.07 g/L^48^. Tanamool et al.^49^ also used the TLC method to screen GABA-producing lactic acid bacteria strains. These authors found that among 44 LAB, the isolates L10-11 clearly produced the highest GABA based on the TLC results^49^. In our results, 14 isolates among 50 LAB were considered as GABA producers. Valenzuela et al.^25^ showed the ability of *Lb. brevis* to produce GABA is consistent with other reported levels for *Lb. brevis* LMG6906 (0.29 g/L). *Lb. brevis* CECT8183 isolated from Spanish cheese by Diana et al.^17^ produced 0.1 g/L of GABA. *Lb. brevis* BJ20 produced 0.002 g/L in a fermented sea tangle solution, which is a popular traditional marine food in Korea based on brown seaweed^50^. *Lb. brevis* PM17 was among the most potent studied isolates by Franciosi et al.^9^. By contrast, no significant GABA production was detected for any *St. thermophilus* strain isolated from yogurt products, compared to lactobacilli and *Lactococcus* species in study. According to the literature, large numbers of strains from these genera from various fermented foods are indicated as GABA-producing LAB^13^. There are many publications reporting variable GABA production levels by different LAB species. Among other factors, production could be affected by the GABA detection methods. For instance, *Lb. delbrueckii* PR1 (63 mg/kg) and *Lac. lactis* PU1 showed the highest GABA concentrations^9^. The results of Ly et al.^10^ showed *Lb. futsaii*, *Lb. namurensis* and *Lb. plantarum* to produce high amounts of GABA in the range of 1.7–2 g/L. Hwang et al.^51^ have examined GABA production by two *Lac. lactis* strains in MRS containing 5% MSG at 30 °C. Maximum GABA production was observed after 40 h (1.37 g/L). Redruello et al.^52^ studied the effect of 6 *Lac. lactis* GABA-producing strains in cheese making. GABA accumulated at concentrations up to 0.457 g/Kg in cheese. Galli et al.^53^ revealed GABA content of their fermented milks by two mix starters (*Lac. lactis* and *Lb. rhamnonus* or *Lb. paracasei*) 0*.*185 and 0.319 g/L, respectively by adding 249 mg/L MSG. Santos-Espinosa et al.^54^ demonstrated the highest GABA concentration (0.086 g/L) in fermented milk with *Lac. lactis* L-571 at 37 °C with 3 g/L of glutamate substrate. In a study conducted by Tajabadi et al.^55^ the optimum conditions for maximum GABA production by *Lb. plantarum* Taj-Apis362 were an initial glutamic acid concentration of 497.97 mM, culture temperature of 36 °C, initial pH of 5.31 and incubation time of 60 h, which produced 0.74 g/L of GABA.

GABA-producing ability is linked to the activity of GAD enzyme in LAB^56^. The source of LAB might affect GAD activity and GABA production capacity, as it was speculated that acidified foods could probably be the natural niche of GABA producers^10^. Li et al.^57^ identified and characterized the GABA-producing lactic acid bacteria and claimed that most LAB strains are able to produce the highest amount of GABA in the range of pH 4 to 5 at 30 to 50 °C and in the presence of glutamic acid. For this reason, this temperature and pH range was used in their study^57^. GABA production occurs under acidic conditions. LAB metabolism mainly leads to the production of organic acids such as lactic and acetic acid. This means that they have to frequently face acidic stress and therefore, they have developed different mechanisms to cope with acidic conditions such as the GAD pathway. Laroute et al.^58^ claimed the activation of this pathway occurred after changing the pH to 4.6 and during the stationary phase. Their results are consistent with our results which optimum pH for GABA production in two examined isolates is 4.5 and 4.6. As reported by Laroute et al.^58^ a dose of 152 mM glutamate, increased the production of GABA by *Lac. lactis* NCDO 2118*.* This dosage of glutamate is similarly optimum dose for GABA production in our examined isolate (*Lac. lactis* 491). The findings of our work showed *Lac. lactis* 311 produced more GABA in lower concentration than *Lac. lactis* 491. The probable reason that lower concentrations of MSG can increase the efficiency of GABA production in comparison to higher concentrations of it, is that large amounts of MSG increase the osmotic pressure of cells and disrupt bacterial metabolism, leading to a decrease in GABA efficiency by bacteria ^59^. Although the optimal concentrations of MSG are different for various microorganisms in GABA production, some researchers have proven that excessive MSG could inhibit cell growth and minimize GABA production^60,61^. Lu et al.^62^ showed that the highest concentration of GABA production by *Lac. lactis* isolated from kimchi was at 34° C (3.68 g/L). These results are similar to our results about *Lac. lactis* 311. However, they stated that the optimal pH of GABA production was in the range of 7–8^62^, which was not in compliance with the results obtained in our study.

## Conclusions

In the present study, firstly, LAB isolates were screened for GABA production according to a qualitative method based on a colorimetric assay. Significant variability in GABA production was encountered among the different species and strains tested. GABA-producing strains were then subjected to a TLC analysis to detect strains with high GABA producing ability. Response surface methodology was finally used to optimize conditions for maximum GABA production in two *Lac. lactis* strains, taking into account the variables affecting the response. The selected strains and the culture conditions producing the highest amounts of this bioactive compound could be used to develop GABA-enriched functional foods or in other biotechnological applications.

## Supplementary Information

Supplementary Information.

## Data Availability

The datasets used and/or analysed during the current study are available from the corresponding author on reasonable request.

## Abbreviations

LAB: Lactic acid bacteria
GRAS: Generally recognized as safe
GABA: Gamma-aminobutyric acid
GAD: Glutamate decarboxylase
NADP: Nicotinamide adenine dinucleotide phosphate
MSG: Monosodium glutamate
TLC: Thin layer chromatography
rep-PCR: Repetitive extra genic profiling PCR
RSM: Response surface methodology
CCD: Central composite design
Rf: Retention factor
