# Supplementary material for: Screening of lactic acid bacteria strains isolated from Iranian traditional dairy products for GABA production and optimization by response surface methodology
Source: Sci Rep. 2023 Jan 9;13:440. doi: 10.1038/s41598-023-27658-5 (PMC9829902; doi:10.1038/s41598-023-27658-5)
Supplement: Supplementary file 1 — Supplementary Information. [file 41598_2023_27658_MOESM1_ESM.pdf]

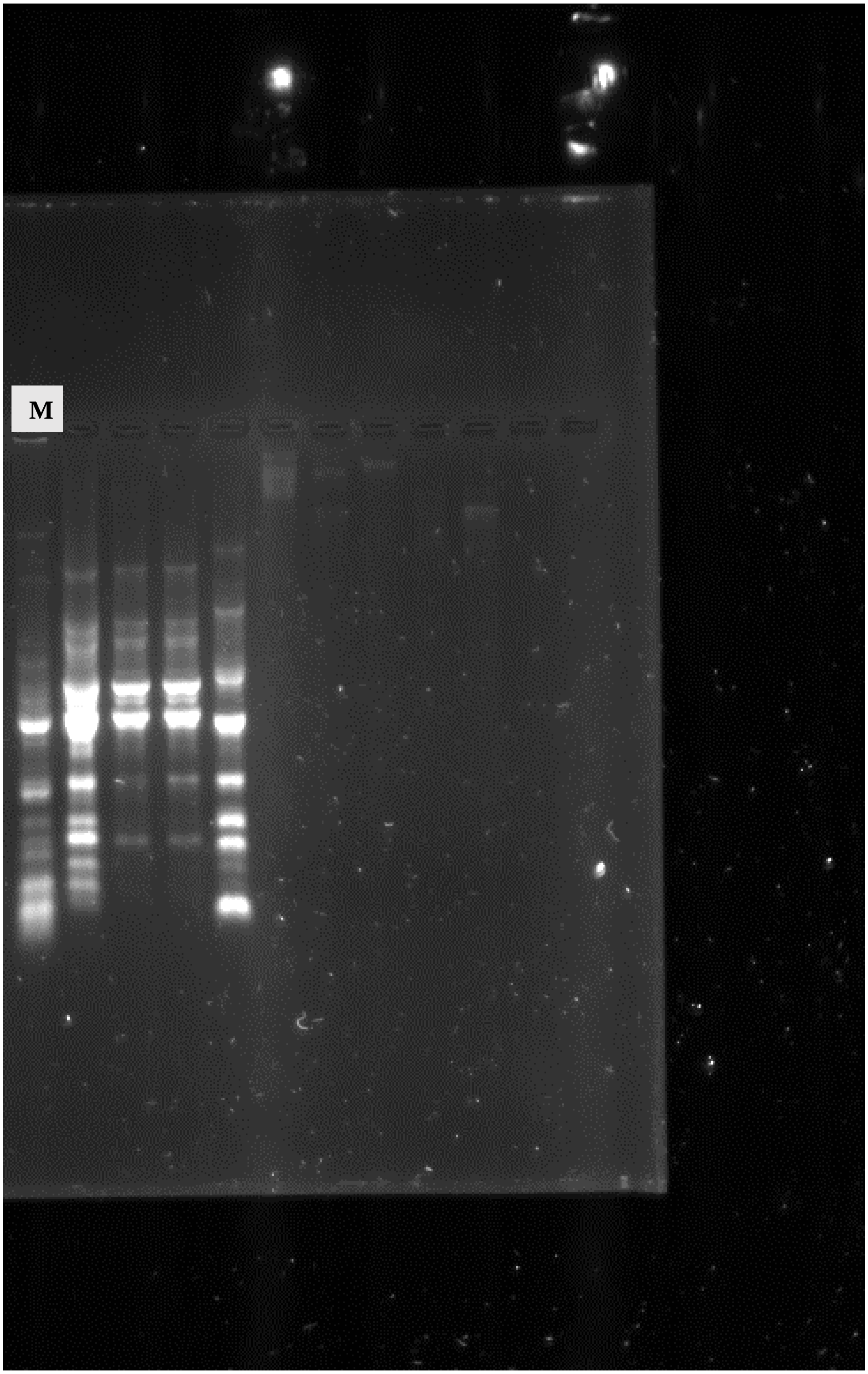

Figure 2 B (Original and un cropped): rep-PCR typing profiles obtained with primer BoxA2R for four *Lactococcus lactis* isolates. First lane: Ladder. M, molecular weight marker.

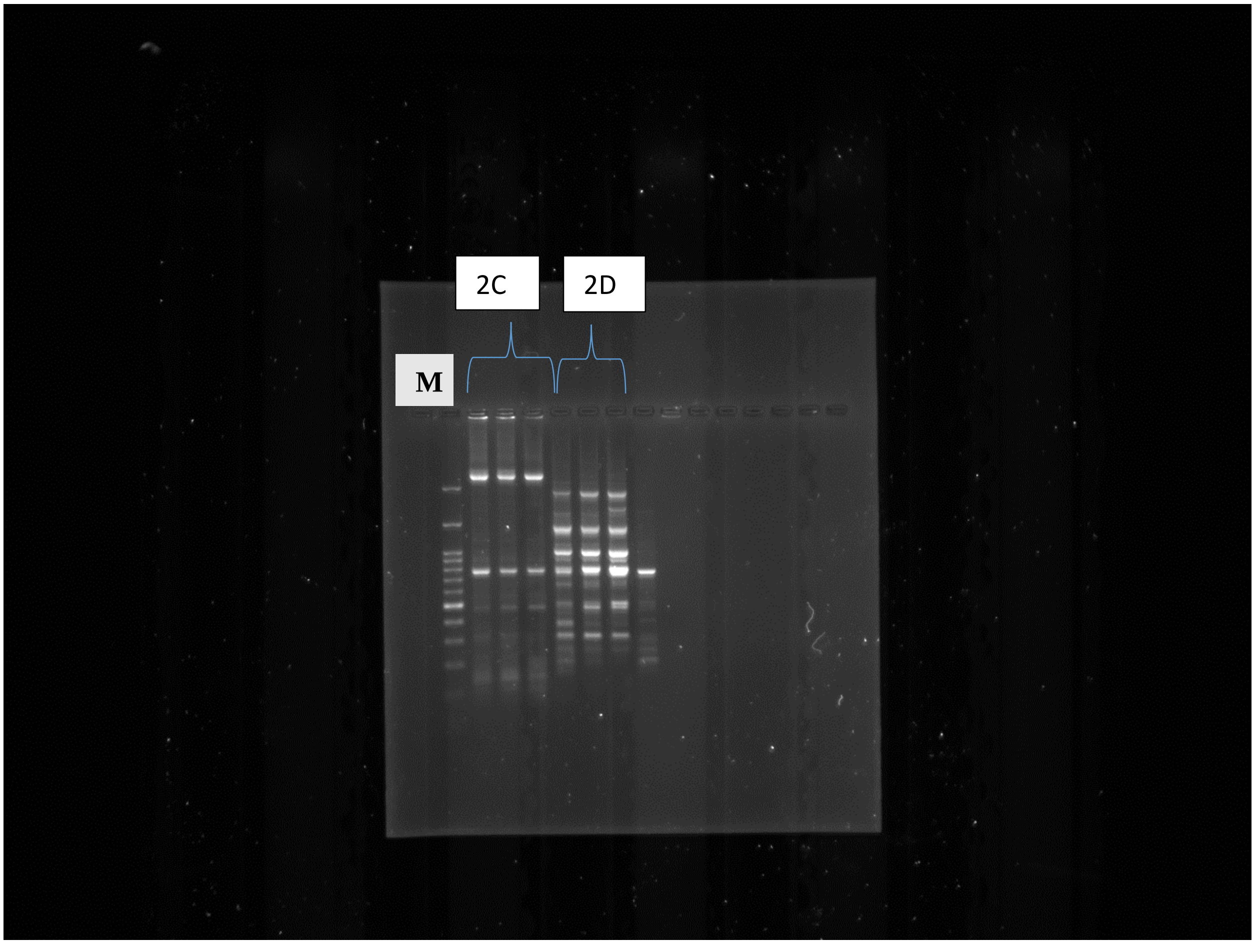

Figure 2C and 2D (Original and un cropped)

Streptococcus.thermophilus (C), and *Lactobacillus delbrueckii* (D)

First lane: Ladder. M, molecular weight marker.
